# Complete Chloroplast Genome Sequence of *Erigeron breviscapus* and Characterization of Chloroplast Regulatory Elements

**DOI:** 10.3389/fpls.2021.758290

**Published:** 2021-11-25

**Authors:** Yifan Yu, Zhen Ouyang, Juan Guo, Wen Zeng, Yujun Zhao, Luqi Huang

**Affiliations:** ^1^School of Food and Biological Engineering, Jiangsu University, Zhenjiang, China; ^2^State Key Laboratory Breeding Base of Dao-di Herbs, National Resource Center for Chinese Materia Medica, China Academy of Chinese Medical Sciences, Beijing, China

**Keywords:** *Erigeron breviscapus*, chloroplast genome sequencing, chloroplast regulatory elements, gene assembly, prokaryotic expression

## Abstract

*Erigeron breviscapus* is a famous medicinal plant. However, the limited chloroplast genome information of *E. breviscapus*, especially for the chloroplast DNA sequence resources, has hindered the study of *E. breviscapus* chloroplast genome transformation. Here, the complete chloroplast (cp) genome of *E. breviscapus* was reported. This genome was 152,164bp in length, included 37.2% GC content and was structurally arranged into two 24,699bp inverted repeats (IRs) and two single-copy areas. The sizes of the large single-copy region and the small single-copy region were 84,657 and 18,109bp, respectively. The *E. breviscapus* cp genome consisted of 127 coding genes, including 83 protein coding genes, 36 transfer RNA (tRNA) genes, and eight ribosomal RNA (rRNA) genes. For those genes, 95 genes were single copy genes and 16 genes were duplicated in two inverted regions with seven tRNAs, four rRNAs, and five protein coding genes. Then, genomic DNA of *E. breviscapus* was used as a template, and the endogenous 5' and 3' flanking sequences of the *trnI* gene and *trnA* gene were selected as homologous recombinant fragments in vector construction and cloned through PCR. The endogenous 5' flanking sequences of the *psbA* gene and *rrn16S* gene, the endogenous 3' flanking sequences of the *psbA* gene, *rbcL* gene, and *rps16* gene and one sequence element from the psbN-psbH chloroplast operon were cloned, and certain chloroplast regulatory elements were identified. Two homologous recombination fragments and all of these elements were constructed into the cloning vector pBluescript SK (+) to yield a series of chloroplast expression vectors, which harbored the reporter gene *EGFP* and the selectable marker *aadA* gene. After identification, the chloroplast expression vectors were transformed into *Escherichia coli* and the function of predicted regulatory elements was confirmed by a spectinomycin resistance test and fluorescence intensity measurement. The results indicated that *aadA* gene and *EGFP* gene were efficiently expressed under the regulation of predicted regulatory elements and the chloroplast expression vector had been successfully constructed, thereby providing a solid foundation for establishing subsequent *E. breviscapus* chloroplast transformation system and genetic improvement of *E. breviscapus*.

## Introduction

*Erigeron breviscapus* (Vant.) Hand-Mazz. is a well-known medicinal plant that belongs to the family Asteraceae, and it is distributed in the southwestern region of China, mainly in Yunnan, Sichuan, Guizhou, and Guangxi Provinces (Li et al., 2013). This herb is commonly used for treating occlusive cerebrovascular disease and sequelae of cerebral hemorrhage and for improving microcirculation, reducing blood viscosity, and preventing platelet aggregation (Li et al., 1998; Sheng et al., 1999; Liu et al., 2010). In addition, *E. breviscapus* has the characteristics of a short growth period, strong vitality, an efficient tissue culture system (Chen et al., 2018), and available genomic information (Jiang et al., 2014; Yang et al., 2017).

However, with the in-depth study of *E. breviscapus* chloroplasts, the current research on the genetic background of this species mainly focuses on the use of the chloroplast genome of *E. breviscapus* for phylogenetic research and germplasm resource protection (Li et al., 2019; Meng et al., 2019), Meng et al. (2019) used the Illumina sequencing platform to obtain a chloroplast genome with a size of 152,183bp, but the article only briefly mentioned the basic structural information of the species’s chloroplast genome and the type and number of genes, and the phylogenetic position of *E. breviscapus* was determined. Li et al. (2019) used the Illumina sequencing platform to obtain a chloroplast genome with a size of 152,367bp, and only briefly introduced the basic structural information of the species’s chloroplast genome and the type and number of genes in the article, and the phylogenetic analysis was conducted to infer phylogenetic position of *E. breviscapus* and *Erigeron multiradiatus* within the family of Asteraceae, the above two reports did not mention the test data and its quality control. Both of the above mentioned studies lacked systematic prediction of the gene structure of the chloroplast genome of *E. breviscapus* and bioinformatics analysis such as codon usage bias analysis and simple sequence repeat (SSR) analysis. In addition, the relevant regulatory elements of the chloroplasts of this species have not been identified. Therefore, for follow-up research on the establishment of the chloroplast genetic transformation system of *E. breviscapus*, it is necessary to obtain a complete *E. breviscapus* cp genome and screen out highly efficient genetic regulatory elements.

Previous studies have reported some chloroplast regulatory elements and proved that these elements play an important role in the level of gene expression. Native genes *trnA/trnI* are located at transcriptionally active spacer regions. After an herbicide resistance gene was integrated into the transcriptionally active spacer region for the first time (Daniell et al., 1998), most subsequent studies preferentially used this site of integration. Compared with the transcriptional silencing spacer (rbcl/accD), the integration of the transgene into the transcriptionally active spacer (trnl/trnA) resulted in 25-fold higher expression of transgenes (Krichevsky et al., 2010) and the *trnA* gene intron included the chloroplast origin of replication and generated more copies of the template (chloroplast vector) to integrate the transgene cassette (Daniell et al., 1990). In addition to the integration site, regulatory sequences located upstream (promoter, 5’UTR) and downstream (3’UTR) of the transgene play a major role in determining its expression level. The most commonly used promoters are photosystem II reaction center promoter PpsbA and *rrn16S* gene promoter Prrn; moreover, the psbA regulatory region, which was first used almost 30years ago (Daniell et al., 1990), still appears to be the best option for use in an expression cassette because the *psbA* gene mediates light-induced activation of translation (Ruhlman et al., 2010) and it also encodes the most highly translated protein in the chloroplast (Klein and Mullet, 1987). In a pioneering work, Fei et al. (2007) studied the *psbB* operon from tobacco chloroplasts and identified a small cistronic element called IEE, which was included in the synthetic structure to drive the expression of *nptII* and *yfp* and proved to be sufficient for mediating polycistronic expression. The polycistronic transcript has been processed into stable and translatable monocistronic mRNA, and this function has been used to express transgenic polycistronic mRNA from a single promoter in a single transformation event (Lu et al., 2013; Bock, 2014; Fuentes et al., 2016). Therefore, for the in-depth study of chloroplast transformation in medicinal plants, it is necessary to further develop and explore the endogenous regulatory elements and establish a more efficient chloroplast transformation system. In this work, we combined second-generation and third-generation sequencing technologies to obtain more completely and accurate *E. breviscapus* cp genome sequence information and performed detailed bioinformatics analysis, including gene annotation, structural statistics, SSR analysis, and codon usage bias analysis. Then, a series of chloroplast regulatory elements were cloned from *E. breviscapus*, and the corresponding chloroplast expression vectors of *E. breviscapus* were constructed and the biological functions of these chloroplast regulatory elements were verified through *in vitro* experiments, thus providing a foundation for the establishment of the subsequent chloroplast genetic transformation system and research on the biology and evolution of *E. breviscapus*.

## Materials and Methods

### Materials and Reagents

Soaked the seeds in deionized water for 3–4h, then placed the seeds at room temperature to fully dry them for 3–4h, soaked the seeds in 1% NaClO solution for 10min in the Vertical Flow Clean Bench, and then washed them with sterile water three times. About 20–30 sterilized seeds were inoculated per petri dish in MS solid medium (Murashige and Skoog, 1962) for germination and then maintained in a culture room under white fluorescent lamps (1,900 lux), with a 16h light/8h dark cycle at 25°C for 4–7days. Individual germinated seedlings were transferred to a sterile tissue culture bottle containing MS medium supplemented with 30g/L sucrose and kept in a culture room under white fluorescent lamps (1,900 lux), with a 16h light/8h dark cycle at 26°C for 4–7weeks.

### Total DNA Extraction and Sequencing

Total genomic DNA was used to build sequence libraries (Illumina Inc., San Diego, CA, United States), and it was extracted from leaves using the cetyltrimethylammonium bromide (CTAB) method. The experimental procedures were performed in accordance with the standard protocol provided by Illumina, and sequence libraries were prepared with the NEB Next Ultra DNA Library Prep Kit. An Illumina NovaSeq sequencer was used to sequence paired-end (PE) sequencing libraries with an average 350bp insert length at the Wuhan Benagen Tech Solutions Company Limited. From these data, over 20 million clean reads with a 150bp read length were passed through quality control. Then, we performed nanopore sequencing to facilitate the connection of high-quality contigs into longer complete sequences in subsequent genome assembly.

### Chloroplast Genome Assembly

Since the original sequencing data may contain low-quality sequences, adaptor sequences, etc. (Ewing and Green, 1998), to ensure the reliability of the information analysis results, the original sequencing data need to be filtered to obtain clean reads, which are stored in FASTQ format (Cock et al., 2009). This project used the filtering software SOAPnuke (version: 1.3.0) to remove reads with an N base content of more than 5%, low-quality (quality value less than or equal to 5) reads with a base number of 50%, and reads with adapter contamination.

UniCycler (0.4.8) software was used to assemble the filtered reads (Wick et al., 2017). First, high-accuracy Illumina data (Q30>85%) were assembled to obtain a high-quality cp genome skeleton (contig), and then nanopore data were used to connect the high-quality contig into a longer complete sequence. The accuracy of the cp genome assembled by this strategy is determined by the accuracy of the Illumina data, and it can effectively avoid the sequence contamination introduced by the error of nanopore data splitting. Finally, Pilon software was used to further correct the Illumina data for errors in the assembled genome, resulting in a genome with higher final accuracy.

Unicycler software was aligned with the close reference genome (NC_043882.1) by blastn (version: BLAST 2.2.30+; parameter: -evalue 1e-5), and the best candidate sequence was selected based on the alignment. The chloroplast genome connection relationship was determined based on the sequence sequencing depth and read alignment and by comparison with closely related species. The connection relationship and whether it forms a loop need to be further experimentally verified.

### Gene Structure Prediction

Chloroplast genome function annotation includes coding gene prediction and noncoding RNA annotation [ribosomal RNA (rRNA) and transfer RNA (tRNA) annotation]. CPGAVAS2^1^ was used for gene annotation and drawn based on the results of the cp genome annotation (Shi et al., 2019).

### Codon Usage Bias Analysis

CodonW (Version: 1.4.4) was used to analyze codon usage bias and statistically estimate the frequency of relative synonymous codon usage (RSCU). Codons with RSCU values >1 were defined as high-frequency codons. We conducted a codon usage bias analysis using the following gene sequences: “ATG”, “TTG”, “CTG”, “ATT”, “ATC”, “GTG”, and “ATA”, which were used as start codons; and “TGA”, “TAG”, and “TAA”, which were used as stop codons; in addition, protein-coding genes shorter than 300bp in length were excluded from the base composition analysis to avoid sampling bias (Wright, 1990).

### Simple Sequence Repeat and Long Repetitive Sequences Analysis

MISA (Sebastian et al., 2017) was used to perform a SSR analysis of the *E. breviscapus* chloroplast [version: 1.0; default parameters: the corresponding minimum number of repetitions of each repeating unit (unit size) are: 1–8, 2–4, 3–4, 4–3, 5–3, and 6–3, with 1–8 meaning that when a single nucleotide is used as the repeat unit, the number of repeats must be at least 8 to be detected; see http://pgrc.ipk-gatersleben.de/misa/misa.html for details].

The software vmatch^2^ was used to find scattered long repetitive sequences in the *E. breviscapus* cp genome (Jean-Simon et al., 2010). The long repeat sequences included three types: forward (F), palindrome (P), and tandem (T) repeats.

### Chloroplast Genome Extraction and PCR Cloning

There is no report on the extraction method of chloroplast of *E. breviscapus* before, therefore, we have developed a method suitable for extracting chloroplast DNA of *E. breviscapus*, which is based on the modified high – salt low – pH method (Shi et al., 2012). Borax was not used in this method, which greatly guaranteed the safety of the operators. In addition, we removed the step of “add 1.5ml of 5mol/L KAc (PH 5.2) and continue freezing for 30min. Then centrifuge at 10,000*g* for 15min, and discard the precipitate”, this saved extraction time and effectively simplified the extraction step. After extracting high-quality chloroplast genomic DNA of *E. breviscapus*, regular PCR was performed as follows: a 30s initial denaturation step at 98°C, followed by 35cycles of denaturation for 10s at 98°C, annealing for 30s at 55–63°C, extension for 30s per kbp sequence length at 72°C, and a final 7min extension step at 72°C. Phusion High-Fidelity DNA Polymerase (New England Biolabs, China) and 2×EasyTaq PCR SuperMix (+dye; TransGen Biotech, China) were utilized for DNA cloning and PCR analysis, respectively. The sequence information of the primers for amplification was provided in Supplementary Table S1. The pEASY-Blunt Zero Cloning Kit (TransGen Biotech, China) was utilized for seamless cloning of the selected amplified fragments.

### Construction of Transforming Vectors

The synthetic operon constructs for chloroplast transformation (pBtEa1t, pBtEa2t, pBtEat3, and pBtEIat) were based on the empty vector pBluescript SK (+). They were all constructed using the pEASY-Uni Seamless Cloning and Assembly Kit (TransGene Biotech, China), which can be used to recombine the vector linearized by any method and the PCR fragment with 15–25bp overlapping region with both ends of the linearized vector. All the fragments needed for chloroplast expression vector construction were then assembled into synthetic operons as follows:

For the first round (Round I) of gene assembly, The empty vector pBluescript SK (+) was digested with Not I and Hind III, and then Eb-PpsbA, *aadA* gene and Eb-TpsbA were cloned into linearized vector and generating construct pBa1, Eb-Prrn, *aadA* gene, and Eb-TrbcL were cloned into linearized vector and generating construct pBa2, Eb-Prrn, *aadA* gene, and Eb-Trps16 were cloned into linearized vector and generating construct pBa3, all the recombinant vectors were verified by Sanger sequencing for the next round of gene assembly.

For the second round (Round II) of gene assembly, the vectors pBa1, pBa2, pBa3, and pBluescript SK (+) were digested with Sal I, and then Eb-PpsbA, *EGFP* gene, and Eb-TpsbA were cloned into linearized vector pBa1 and generating construct pBEa1, Eb-Prrn, *EGFP* gene, and Eb-TrbcL were cloned into linearized vector pBa2 and generating construct pBEa2, Eb-Prrn, *EGFP* gene, and Eb-Trps16 were cloned into linearized vector pBa3 and generating construct pBEa3, Eb-Prrn, *EGFP* gene, Eb-IEE, *aadA* gene, and Eb-Trps16 were cloned into linearized vector pBluescript SK (+) and generating construct pBEIa, all the recombinant vectors were verified by Sanger sequencing for the next round of gene assembly.

For the third round (Round III) of gene assembly, the vectors pBEa1, pBEa2, pBEa3, and pBEIa were digested with Kpn I, and then the homologous recombination fragment Eb-trnI was cloned into linearized vectors and generating construct pBtEa1, pBtEa2, pBtEa3, and pBtEIa, respectively and verified by Sanger sequencing. Finally, the vectors pBtEa1, pBtEa2, pBtEa3, and pBtEIa were digested with Sac I, and then the homologous recombination fragment Eb-trnA was cloned into linearized vectors and generating construct pBtEat1, pBtEat2, pBtEat3, and pBtEIat (Supplementary Figure S1) and verified by Sanger sequencing.

### Analysis of *aadA* Gene Expression in *E. coli*

After PCR identification and Sanger sequencing, four verified vectors (pBtEat1, pBtEat2, pBtEat3, and pBtEIat) and the empty vector pBluescript SK (+) were transformed into the *E. coli* expression strain Transetta (DE3). Then, the transformed *E. coli* strains were grown in Luria-Bertani (LB) medium at 37°C unless otherwise indicated. Spectinomycin and ampicillin were used for selection at appropriate concentration.

### Gene Expression Analysis of *EGFP*

After culturing in 20ml of LB medium for 14–16h at 16°C (150–180rpm), *EGFP* gene expression in transformed *E. coli* strains was examined using a Thermo Scientific Varioskan LUX Multimode Microplate Reader (Thermo Fisher Scientific, MA, United States). The loading volume was 200μl, and the “Fluorescence” protocol was executed in Skanlt Software 6.1 RE, which included an excitation wavelength at 485nm and an emission wavelength at 520nm. Subsequently, the fluorescence intensity of *E. coli* strains containing different vectors was counted. Each sample was repeated for three times to ensure the credibility of the data, in order to compare the effects of different combinations of chloroplast regulatory elements on the expression level of green fluorescent protein in *E. coli*, the expression level of fluorescent protein in each sample was compared by unpaired *t*-test (*p*<0.05), the results are represented as a bar graph.

### Statistical Analysis

The statistical evaluation was performed using SPSS 19.0 software (IBM Corp., United States). All data were presented as the means±SE of at least three replicates. ANOVA was followed by a *t*-test. Mean values were considered significantly different at *p*<0.05.

## Results

### Chloroplast Assembly and Genome Features

This project adopted the whole genome shotgun strategy to construct a 350bp library from the DNA samples of *E. breviscapus* and then used the Illumina NovaSeq sequencer to perform PE sequencing on this library, and the reads were 150bp in length. In this sequencing, the raw data sequencing volume of the *E. breviscapus* sample was 4.2Gbp. After data filtering, the clean data volume of the sample was 4.1Gbp (Table 1). The original sequencing statistics of the third-generation nanopore sequencing were shown in Supplementary Table S2.

**Figure 1:**
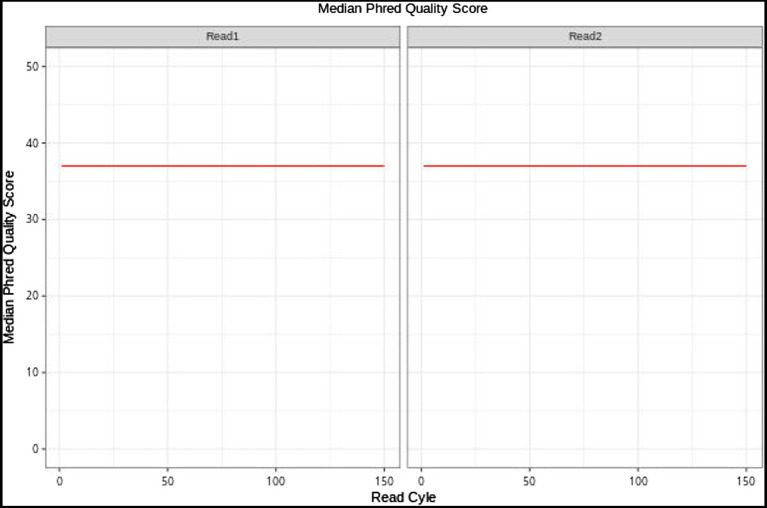
Base quality value distribution graph of *E. breviscapus* sample. The graph reflects the average sequencing quality (Y-axis) of read 1 and read 2 along the Read Cycle (X-axis).

The *E. breviscapus* cp genome was completely assembled into a single molecule of 152,164bp, the assembly result of the cp genome of *E. breviscapus* contained 0 Gap, the GC content was 37.20, the assembly coverage was 332.55X, and the sequencing quality value (Phred) was above 30 (Figure 1), indicating that the sequencing quality was high.

**Table 1:** Sequencing data volume information statistics.

Sample name	Length (bp)	Raw reads	Raw bases (bp)	Clean reads	Clean bases (bp)	GC (%)	Q20 (%)	Q30 (%)
*Erigeron breviscapus*	150	27,679,808	4,151,971,200	27,467,550	4,120,132,500	37.73	98.55	95.37

The conserved size and quadripartite structure of the complete *E. breviscapus* cp genome was similar to the cp genomes of previously published data. These genomes were usually 152kb in size and included two inverted repeat regions (IRs) and two single-copy regions, namely, a large single copy and small single copy (LSC and SSC, respectively) region.

**Figure 2:**
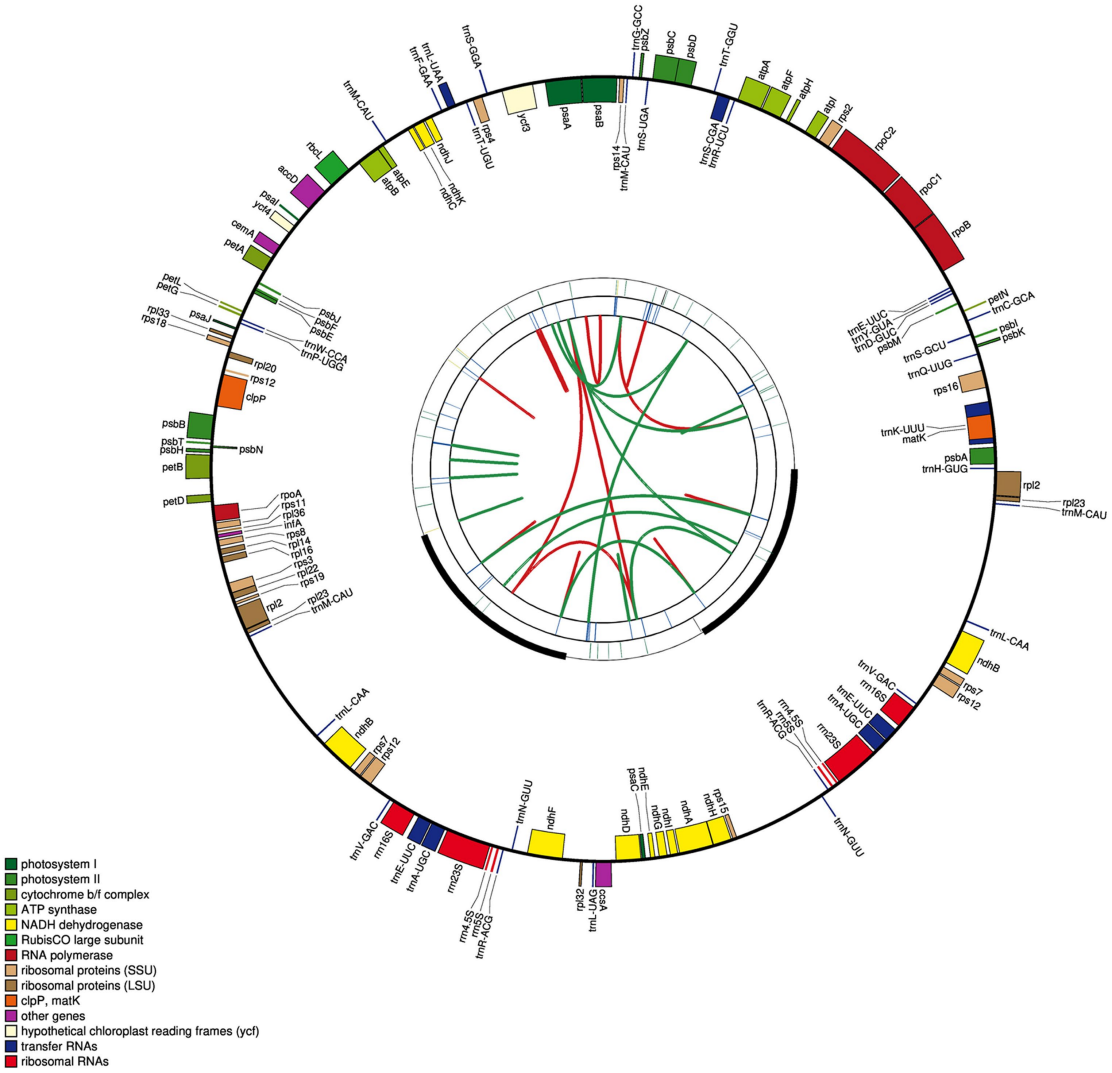
Circular map of the *E. breviscapus* cp genome. There are four rings on the cp genome map. From the center to the outside, the first circle shows the forward and reverse repetitions connected by the red arc and the green arc respectively; The second circle shows the tandem repeat marked with a short bar; The third circle is the microsatellite sequence identified by MISA; the fourth circle was drawn with genemap to show the gene structure on the plastids, Genes shown outside and inside of the fourth circle are transcribed counterclockwise and clockwise, respectively; the colors of genes are distinguished according to their functional classification.

The cp genome of *E. breviscapus* consisted of two single-copy regions isolated by two identical IR regions of 24,699bp each, one LSC region of 84,657bp and one SSC region of 18,109bp. The proportions of LSC, SSC, and IR sizes in the entire cp genome were 55.6, 11.9, and 32.5%, respectively (Figure 2; Table 2). The GC contents of the LSC, SSC, and IR and whole cp genome were 35.06, 31.00, 43.13, and 37.20%, respectively, which were consistent with the published *E. breviscapus* cp genomes (Supplementary Table S3). The cp genome of *E. breviscapus* was compared to previously published data, no structural differences were found among all compared data, it demonstrated that gene order, gene content, and entire genome structure were conserved in *E. breviscapus* cp genome.

**Table 2:** Chloroplast structure statistics of *Erigeron breviscapus* samples.

Region	Start	End	Length	A (%)	T (%)	C (%)	G (%)	AT (%)	GC (%)
Total genome	1	152,164	152,164	31.36	31.45	18.54	18.65	62.80	37.20
LSC	1	84,657	84,657	32.29	32.65	17.25	17.80	64.94	35.06
IRA	84,658	109,356	24,699	28.37	28.50	20.73	22.40	56.87	43.13
SSC	109,357	127,465	18,109	34.97	34.03	16.33	14.67	69.00	31.00
IRB	127,466	152,164	24,699	28.50	28.37	22.40	20.73	56.87	43.13

### Gene Content and Structure

The cp genome of *E. breviscapus* consisted of 127 coding regions made up of 83 protein-coding genes, 36 tRNAs, and eight rRNAs, of which 95 genes were unique and 16 genes were repeated in two inverted regions consisting of five protein coding genes, seven tRNAs, and four rRNAs (Figure 2; Table 3). Among these 95 unique genes, one gene crossed different cp genome boundaries: *rps12* crossed two IR regions, the LSC region and the SSC region (two 3′ end exons repeated in IRs and 5′ end exon situated in LSC; Figure 2). Of the remaining 94 genes, 82 were situated in the LSC, including 61 protein-coding genes and 21 tRNAs, and 12 were situated in the SSC, including 11 protein-coding genes and one tRNA.

**Table 3:** List of genes in the *E. breviscapus* cp genome.

Gene group	Gene name
NADH dehydrogenase	*ndh*, *ndhI*, *ndhJ*, *ndhK*, *ndhA*, *ndhH*, *ndhD*, *ndhC*, *ndhB*, *ndhG*, and *ndhE*
Other genes	*ccsA*, *cemA*, and *accD*
RNA polymerase	*rpoC2*, *rpoA*, *rpoB*, and *rpoC1*
Transfer RNAs	*trnS-GCU*, *trnD-GUC*, *trnH-GUG*, *trnM-CAU*, *trnR-ACG*, *trnF-GAA*, *trnY-GUA*, *trnP-UGG*, *trnT-GGU*, *trnS-GGA*, *trnL-UAA*, *trnC-GCA*, *trnV-GAC*, *trnG-GCC*, *trnL-CAA*, *trnS-UGA*, *trnQ-UUG*, *trnR-UCU*, *trnS-CGA*, *trnA-UGC*, *trnL-UAG*, *trnT-UGU*, *trnE-UUC*, *trnN-GUU*, *trnK-UUU*, and *trnW-CCA*
Ribosomal proteins (SSU)	*rps18*, *rps19*, *rps2*, *rps7*, *rps16*, *rps15*, *rps8*, *rps11*, *rps12*, *rps4*, *rps3*, and *rps14*
Maturase	*matK*
Cytochrome b/f complex	*petA*, *petL*, *petG*, *petB*, *petD*, and *petN*
Photosystem II	*psbK*, *psbT*, *psbB*, *psbH*, *psbD*, *psbF*, *psbZ*, *psbC*, *psbM*, *psbA*, *psbN*, *psbJ*, *psbI*, and *psbE*
ATP synthase	*atpI*, *atpA*, *atpE*, *atpF*, *atpB*, and *atpH*
Photosystem I	*psaJ*, *psaC*, *psaB*, *psaA*, and *psaI*
RubisCO large subunit	*rbcL*
Ribosomal proteins (LSU)	*rpl20*, *rpl32*, *rpl23*, *rpl33*, *rpl16*, *rpl2*, *rpl14*, *rpl36*, and *rpl22*
Translational initiation factor	*infA*
Ribosomal RNAs	*rrn23S*, *rrn16S*, *rrn5S*, and *rrn4.5S*
Protease	*clpP*
Hypothetical chloroplast reading frames (ycf)	*ycf2*, *ycf3*, and *ycf4*

The *E. breviscapus* cp genome was composed of protein coding genes, tRNAs, rRNAs, and intronic and intergenic regions. Noncoding DNA accounted for 68,539bp (45.0%) of the whole *E. breviscapus* cp genome, protein-coding genes accounted for 71,850bp (47.2%), tRNA accounted for 2,725bp (1.8%), and rRNA accounted for 9,050bp (6.0%).

Most of the protein-coding genes contained only one exon, while 13 genes contained at least one intron, of which eight genes were distributed in the LSC, one was distributed in the SSC and four were distributed in both IRs (Supplementary Table S4). Among them, three genes (*rps12*, *clpP*, and *ycf3*) contained two introns, while 10 genes (*trnA-UGC*, *trnE-UUC*, *trnL-UAA*, *rpl2*, *ndhA*, *ndhB*, *petB*, *atpF*, *rpoC1*, and *trnK-UUU*) contained one intron. The longest intron of *trnK-UUU* was 2,543bp, and it included the 1,515bp encoding the *matK* gene (Gu et al., 2016). The *rps12* gene was predicted to be trans-spliced with a repeated 3′ end duplicated in the two IRs and a single 5′ end exon in the LSC (Redwan et al., 2015).

### Statistics of Codon Usage Bias

Relative synonymous codon usage is an assessment of synonymous codon usage bias. This value is equal to the ratio of the actual observed value of synonymous codons to the expected value of the average use of synonymous codons. If there is no bias for codon usage, then the RSCU value will be 1; if the codon is used more frequently than other synonymous codons, then its RSCU value will be greater than 1; otherwise, the RSCU value will be less than 1 (Nie et al., 2012; Liu et al., 2020).

The results of the RSCU value analysis (Supplementary Table S5) of various amino acids showed that there were 31 codons with RSCU≥1, of which 12 codons, such as UUA, AGA, CAA, and GGA, end in A; 16 codons, such as UCU, GCU, ACU, and UAU, end in U; and the only ones ending with G are UUG, UGG, and AUG. These findings indicated that the codons ending with A and U in the chloroplast genome of *E. breviscapus* appeared more frequently and are preferred codons, while the codons ending with C and G were non-preferred codons.

### Chloroplast Genome Simple Sequence Repeats

Simple sequence repeats are sequences with motifs from 1 to 6bp in length repeated multiple times (see section “Materials and Methods” for cutoff criteria), and they are distributed throughout the cp genome and often used as markers for breeding studies, population genetics, and genetic linkage mapping (Grassi et al., 2002; Timme et al., 2007).

**Figure 3:**
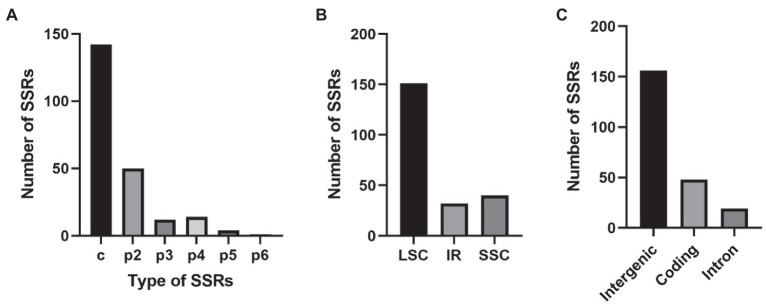
The type, distribution, and presence of simple sequence repeats (SSRs) in *E. breviscapus* cp genome. **(A)** Number of different SSR types detected in *E. breviscapus* cp genome. **(B)** Frequency of SSRs in the large single copy (LSC), small single copy (SSC), and inverted repeat (IR) regions. **(C)** Frequency of SSRs in the protein-coding regions, intergenic spacers, and intron.

A total of 223 SSRs were found in the *E. breviscapus* cp genome (Figure 3; Supplementary Table S6). These SSRs included 142 complex repeat-type SSRs (63%), 50 dinucleotide SSRs (22%), 12 trinucleotide SSRs (5%), 14 tetranucleotide (6%), four pentanucleotide SSRs (1%), and one hexanucleotide SSR (0.003%; Figure 3A; Supplementary Table S6). Among the 223 SSRs, 87% of SSRs (195) were the AT type, with copy numbers from 8 to 23 (Supplementary Table S6). Forty-eight SSRs were detected in protein-coding genes, 19 SSRs were detected in introns, and 156 were detected in intergenic regions (Figure 3C). In relation to the quadripartite, 151 SSRs were situated in the LSC, while 32 and 40 were identified in the IR and SSC, respectively (Figure 3B).

### Identification of Long Repetitive Sequences

Long repetitive sequences may promote the rearrangement of the cp genome and increase population genetic diversity (Cavalier-Smith, 2002). A total of three unique repeats were found in the *E. breviscapus* cp genome, the size of two palindromic repeats were 48bp and 24,699bp, and the forward repeat was 39bp in size. The palindromic repeat which was 48bp in size had two same starting sites in the chloroplast genome sequence, these two starting sites were located at 74,576bp, another palindromic repeat’s two starting site were located at 84,657 and 127,465bp respectively, the first starting site of the forward repeat located at 43,603bp and the second starting site located at 119,435bp. In addition, forward repeats are usually caused by transposon activity (Gemayel et al., 2012), which will increase under cell stress (Voronova et al., 2014). However, the origin and production mechanism of long repetitive sequences are not yet fully understood. Previous studies have shown that slipped-strand mispairing and inapposite recombination of repetitive sequences may lead to genome rearrangement (Timme et al., 2007). Moreover, forward repeats can cause changes in genome structure; therefore, they can be used as markers in phylogenetic studies.

### Cloning of Chloroplast Regulatory Elements of *E. breviscapus*

Before cloning the target sequence, we first obtained the specific sequence information of the target sequence through previous research, and this information included the sequence length, putative transcriptional elements, and secondary structure. Then, through a sequence alignment, secondary structure analysis, etc., the specific information of these target sequences was initially obtained from the cp genome of *E. breviscapus* (Table 4).

**Table 4:** Regulatory elements of *E. breviscapus* cp genome.

Feature	Type	Size (bp)	Transcriptional elements	
Eb-trnI	Site of integration	1,322	-	
Eb-trnA	Site of integration	936	-	
Eb-Prrn	Promoter+rbcL5'UTR	135	−10 consensus sequence	TATATT
			−35 consensus sequence	TTGACG
			GAA-box	GAA
			SD-like sequence	GGAGG
Eb-PpsbA	Promoter+psbA5'UTR	244	−10 consensus sequence	TATACT
			−35 consensus sequence	TTGACA
			Ribosome binding site 1	AAG
			Ribosome binding site 2	TGATAAT
			AU-box	TAAATAAA
Eb-TpsbA	3'UTR	138	Stem-loop	
Eb-TrbcL	3'UTR	309	Stem-loop	
Eb-Trps16	3'UTR	303	Stem-loop	
Eb-IEE	IEE+rbcL5'UTR	67	Stem-loop	-
			S-box	AAGTCAA
			SD-like sequence	GGAGG

**Figure 4:**
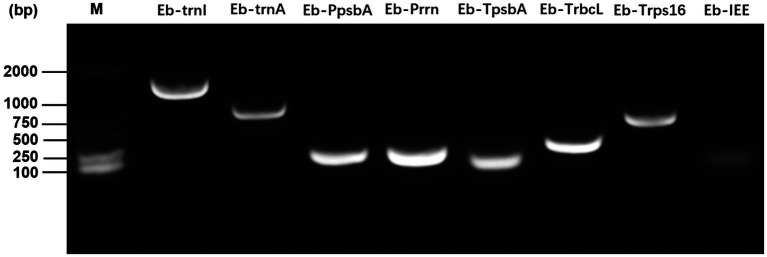
PCR amplification of chloroplast regulatory elements of *E. breviscapus*.

Using *E. breviscapus* genomic DNA as a template, the target gene fragment was amplified with the primers shown in Supplementary Table S1. As shown in Figure 4, all amplified bands were the same size as the target gene fragment. Eb-Prrn was a fragment containing the rRNA operon promoter from *E. breviscapus* which was PCR amplified with a Shine-Dalgarno (SD) sequence derived from the chloroplast *rbcL* gene, Eb-psbA was a fragment containing the *E. breviscapus* psbA promoter and the *E. breviscapus* psbA leader. Eb-TpsbA, Eb-TrbcL, and Eb-Trps16 were the terminator of *psbA*, *rbcL* and *rps16* gene, respectively. Eb-IEE was an intercistronic expression element conferring intercistronic RNA processing and, in this way, enhancing expression of downstream cistrons of the operon. Eb-trnI was a fragment containing the *trnI* gene and part of the flanking sequence of this gene and Eb-trnA was a fragment containing the *trnA* gene and part of the flanking sequence of this gene. The cloned chloroplast regulatory element sequence can be used for subsequent vector construction.

### Analysis of *aadA* Gene Expression in *E. coli*

**Figure 5:**
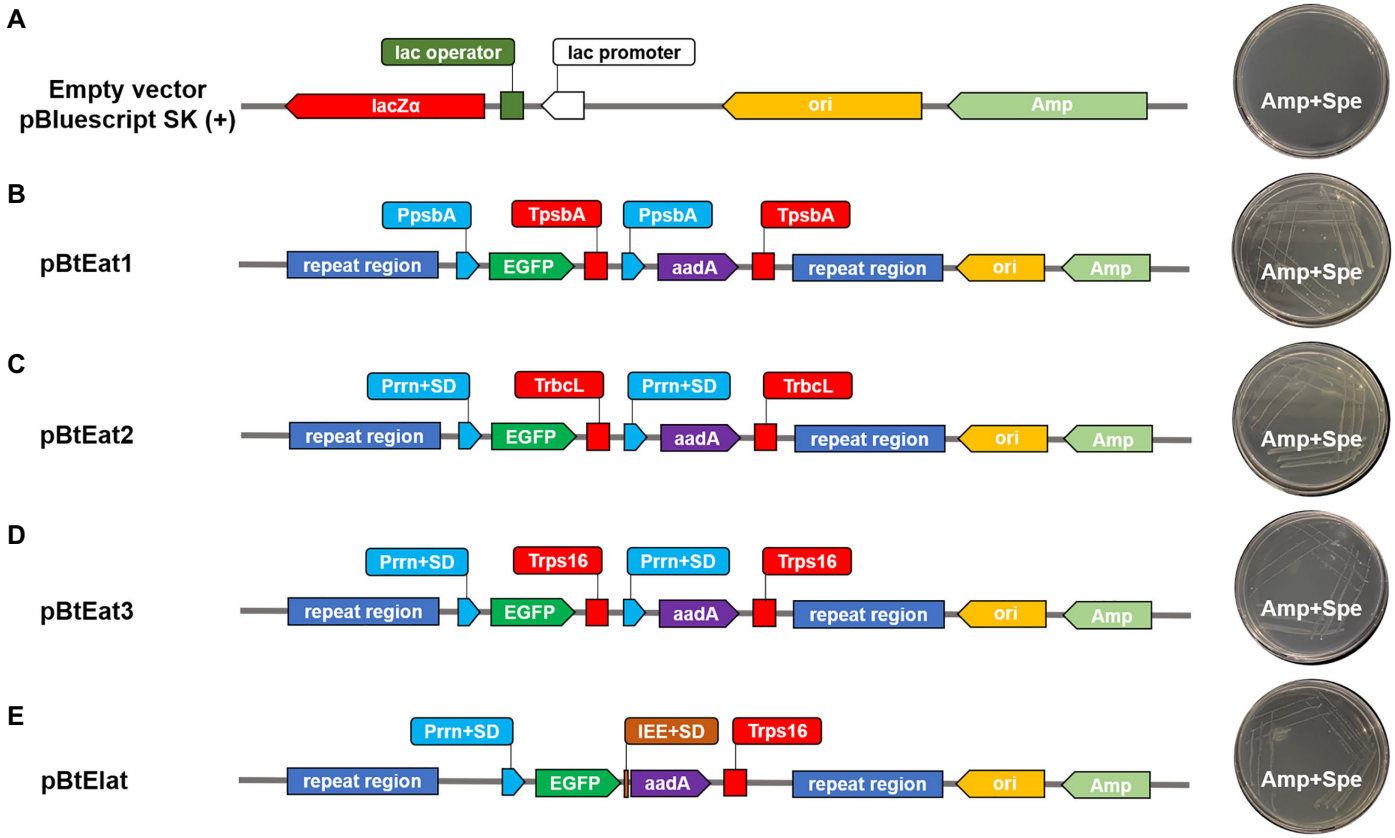
The expression of *aadA* gene in *Escherichia coli*. **(A)**: *Escherichia coli* containing empty vector, **(B–E)**: *Escherichia coli* containing recombinant vector.

The verified vectors pBtEat1, pBtEat2, pBtEat3, and pBtEIat were transformed into the *E. coli* expression strain Transetta (DE3). As shown in Figure 5, after incubation, *E. coli* containing the empty vector could not grow on LB medium containing ampicillin and spectinomycin (Figure 5A). However, *E. coli* containing the recombinant vector could be grown on LB medium containing ampicillin and spectinomycin (Figures 5B–E), indicating that the selected regulatory elements had prokaryotic characteristics and could mediate the correct expression of foreign genes.

### Detection of *EGFP* Expression

**Figure 6:**
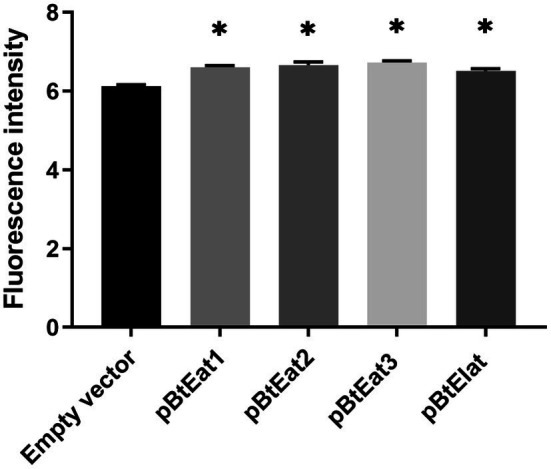
The expression level of *EGFP* gene in *E. coli*. Data are presented as mean±SE (*n*=3). The asterisk represents a significant difference (^*^p<0.05).

As shown in Figure 6, the fluorescence intensity of the *E. coli* expression strain containing the recombinant vector was higher than that of the control test group. Among them, the expression strain containing the recombinant vector pBtEat3 (containing the Prrn-EGFP-Trps16 expression cassette) had the highest fluorescence intensity, which showed that the promoters Prrn and 3′ UTR Trps16 could mediate the high-efficiency expression of foreign genes in *E. coli*. In this study, the *EGFP* gene was efficiently expressed in *E. coli* transformed with the *E. breviscapus* chloroplast expression vector, suggesting that the *EGFP* gene may also be highly expressed in *E. breviscapus* chloroplasts.

## Discussion

The present work was to use high-throughput sequencing technology to systematically and comprehensively analyze the chloroplast genome of *E. breviscapus*. On this basis, we used the prokaryotic expression system to screen out high-efficiency chloroplast regulatory elements, which laid the foundation for the subsequent establishment of the chloroplast genetic transformation system of *E. breviscapus*.

Although, there have been reported on the chloroplast genome of *E. breviscapus*, since the *E. breviscapus* used in this study is cultivated species, It is difficult to rule out differences in genetic background between cultivated species and other varieties. In order to have a clearer understanding of the genetic background of the chloroplast of *E. breviscapus* used in this article, as well as to provide an accurate reference for the selection of chloroplast regulatory elements and the efficient expression of foreign genes for chloroplast transformation, we combined second-generation and third-generation sequencing technologies, the advantage of this method is the long read lengths (Eid et al., 2009), which facilitate *de novo* genome assembly, particularly in the four chloroplast junctions between the single-copy regions and IR regions. At the same time, using the Illumina short read length can also improve the low accuracy of the long reads generated by the nanopore platform (~85% of the raw data; Redwan et al., 2015). The chloroplast genome assembly statistics and bioinformatics analysis data in this article were compared with the previously published data. It was obviously that the chloroplast genome of *E. breviscapus* reported in the three articles were conserved in gene order, gene content, and entire genome structure, but previous reports only provided a little bit of genome information and have not provided a more systematic and comprehensive bioinformatics analysis of the chloroplast genome of *E. breviscapus*. Therefore, we conducted an in-depth analysis of chloroplast genome data of *E. breviscapus*, including a systematic classification of chloroplast genes, SSR analysis, codon usage bias analysis, long repetitive sequences analysis, and this process allowed us to further understand the genomic structure and genetic background of the chloroplast of *E. breviscapus*. On this basis, we successfully screened the target chloroplast regulatory elements from the cp genome of *E. breviscapus* through an extensive literature research and accurate bioinformatics analysis. Active promoters with suitable 5′ UTR and 3′ UTR are important constitutions of efficient transformation and expression vectors. It is generally thought that the use of these endogenous genes can greatly promote the integration and expression of foreign genes in the host genome (Radakovits et al., 2010). In this work, two homologous recombinant fragments, two high-efficiency promoters, three 3′ UTR sequences, one SD sequence and one intercistronic expression element were identified from the chloroplasts of *E. breviscapus*.

Sequence analysis was performed on the chloroplast regulatory elements for prediction of its potential cis-acting elements involved with transcription initiation, regulation, or termination. In the Eb-PpsbA and Eb-Prrn sequence, −10 consensus sequence, and -35 consensus sequence were detected. The −10 consensus sequence is located at the core promoter region and involved with accurate transcription initiation. The −35 consensus sequence commonly exists in many prokaryotic promoters, which acts as an enhancer region. Translation initiation occurs on a specific sequence called the ribosome binding site, this is a short base sequence located in front of the coding region. This site can be complementary to the 3′ end of the ribosomal 16S rRNA, which promotes the binding of the ribosome to the mRNA and facilitates the initiation of translation. Two ribosome binding sites were identified from Eb-PpsbA, since Eb-Prrn lacks a ribosome binding site, we added a ribosome site from the *rbcL* gene of *E. breviscapus* chloroplast at the 3′ end of Eb-Prrn by PCR amplification to ensure that the sequence can have transcription and translation initiation function. The 3′ UTR sequence of the prokaryotic expression system can ensure the stability of mRNA and facilitate the accumulation of protein level, these 3′ UTR sequences usually contain a stable stem-loop structure (Monde et al., 2000). Through, the RNA secondary structure prediction analysis of Eb-TpsbA, Eb-TrbcL, and Eb-Trps16, we found that they all have a stable stem-loop structure. Before this report, IEE for the stacking and expression of foreign genes in the chloroplast of *E. breviscapus* had not been identified. Therefore, we preliminarily screened out the endogenous IEE sequence of *E. breviscapus* through sequence comparison and applied it to the construction of the polycistronic expression cassette of the vector.

To ensure that the selected regulatory elements have the correct biological functions, we have further identified these chloroplast regulatory elements through the prokaryotic expression system. The most effective selectable marker used in chloroplast transformation is the *aadA* gene, which confers resistance to streptomycin and spectinomycin. In this study, the transformed *E. coli* can grow on LB medium containing spectinomycin. The result indicated that these regulatory elements could regulate gene expression in *E. coli* and had the correct prokaryotic expression characteristics. Finally, we used a microplate reader to determine the accumulation level of green fluorescent protein *in vitro*, and compared the expression efficiency of different combinations of regulatory elements by fluorescence intensity. Compared with the empty vector, all the transformed *E. coli* exhibited extremely high fluorescence intensity, suggesting that Eb-Prrn and Eb-PpsbA had priming activity, which ensured the efficient transcription of the *EGFP* gene. In addition, the 5′ UTR regions and ribosome binding sites contained in these two sequences also promote the translation of green fluorescent protein in *E. coli*; Eb-TpsbA, Eb-TrbcL, and Eb-Trps16 can also promote the accumulation of green fluorescent protein. Comparing the fluorescence intensity of pBtEat2 and pBtEat3 strains, we found that different 3′ UTR sequences (Eb-TrbcL and Eb-Trps16) could cause different accumulation level of green fluorescent protein when *EGFP* gene was driven by Eb-Prrn. The pBtEIat strain could be grown on LB medium with spectinomycin and could be detected with green fluorescence. These results indicated that Eb-IEE could active the expression of *EGFP* gene and *aadA* gene as well as polycistronic expression. Notably, the pBtEat3 strain had the highest fluorescence intensity, which indicated that the combination of Eb-Prrn and Eb-Trps16 had higher transcription and translation efficiency. This combination could be used for subsequent chloroplast transformation system, which is the preferred choice for vector construction.

In conclusion, this study provided complete information on the cp genome of *E. breviscapus* and identified a series of chloroplast expression elements with prokaryotic expression characteristics, and these findings can provide a foundation for the establishment of a chloroplast genetic transformation system of *E. breviscapus*.

## Data Availability Statement

The data presented in the study are deposited in the GenBank repository, accession number: OK524211 (Eb-trnI), OK524212 (Eb-trnA), OK524213 (Eb-Prrn), OK524214 (Eb-PpsbA), OK524215 (Eb-Trps16), OK524216 (Eb-TpsbA), OK524217 (Eb-TrbcL), and OK524218 (Eb-IEE).

## Author Contributions

YY and YZ wrote the manuscript. YY, YZ, ZO, JG, and WZ performed the experiments. LH and YZ supervised the research. All authors contributed to the article and approved the submitted version.

## Funding

This work was supported by the National Key R&D Program of China (2020YFA0908000), the National Natural Science Foundation of China (81891013), and the Key Project at Central Government Level: The ability establishment of sustainable use for valuable Chinese Medicine Resources (2060302) to LH, and the Fundamental Research Funds for the Central public welfare research institutes (ZZXT202103) to YZ.

## Conflict of Interest

The authors declare that the research was conducted in the absence of any commercial or financial relationships that could be construed as a potential conflict of interest.

## Publisher’s Note

All claims expressed in this article are solely those of the authors and do not necessarily represent those of their affiliated organizations, or those of the publisher, the editors and the reviewers. Any product that may be evaluated in this article, or claim that may be made by its manufacturer, is not guaranteed or endorsed by the publisher.

## Footnotes

^1^ http://47.96.249.172:16019/analyzer/annotate

^2^ http://www.vmatch.de/
