# Supplementary material for: Complete Chloroplast Genome Sequence of Erigeron breviscapus and Characterization of Chloroplast Regulatory Elements
Source: Front Plant Sci. 2021 Nov 25;12:758290. doi: 10.3389/fpls.2021.758290 (PMC8657942; doi:10.3389/fpls.2021.758290)
Supplement: Supplementary file 1 [file Table_1.docx]

**Supplementary Table 1.** Primers for all PCR.

| **Primer** | **Sequence (5’ to 3’)** |
| --- | --- |
| EGFP-F | ATGGTGAGCAAGGGCGAGGA |
| EGFP-R | TTAAAGATCTTCTTCAGAAATCAACTTTTGTTC |
| t(aadA)-F | ATGGGGGAAGCGGTGATC |
| aadA-R | TTATTTGCCGACTACCTTGGTGAT |
| pBtrnI-recom-F | TATAGGGCGAATTGGGTACCGGGCCTTGTACACACCGC |
| pBtrnI-recom-R | TCGAGGGGGGGCCCGGTACCATTCTTTTCCCTGGCGCA |
| pBtrnA-recom-F | TAGAGCGGCCGCCACCGCGGGCTCCACTTGGCTCGGGG |
| pBtrnA-recom-R | AAGCTGGAGCTCCACCGCGGAGCTTTGTATCGGCTAAGTTCACG |
| pBEPpsbA-F | GGCCCCCCCTCGAGGTCGACTGTCATAGTTAATTAAATTCTATTGTCTGTATCTG |
| pBEPpsbA-R | TCCTCGCCCTTGCTCACCATGGTAAAATCTTGGTTTATTTAATTATCAGG |
| pBETpsbA-F | TTTCTGAAGAAGATCTTTAAGACTTTGGTCTGATTGTATTGTATAGGA |
| pBETpsbA-R | AGCTTATCGATACCGTCGACTAGTATAGTATTACTATATAATTTTTTTCCATTAACAT |
| pBEPrrn-F | GGCCCCCCCTCGAGGTCGACAACCCAATGTGAGTTTTTCATTTTG |
| pBEPrrn-R | TCCTCGCCCTTGCTCACCATTTCATAGTTGCATTACTTATAGCTTCCTT |
| pBETrbcL-F | TTTCTGAAGAAGATCTTTAACGTTCGTTCTCTTAATTGAATTTCA |
| pBETrbcL-R | AGCTTATCGATACCGTCGACACAATATACAGGATGGGTAGAAAAAGTT |
| pBETrps16-F | TTTCTGAAGAAGATCTTTAATCAAACAAAATTAAATTAAGGAAATAAAAAAAG |
| pBETrps16-R | AGCTTATCGATACCGTCGACATGGAATCCATATAGAATAAAGAACTTCTATTC |
| pBEIEE-F | TTTCTGAAGAAGATCTTTAATAAGATCGTTTATTTACAATGGGA |
| pBaIEE-R(SD) | GCGATCACCGCTTCCCCCATAAATCCCTCCCTACAACTTGAAATCTGTTGACTTTGTATACC |
| pBaPpsbA-F | TCGACGGTATCGATAAGCTTTGTCATAGTTAATTAAATTCTATTGTCTGTATCTG |
| pBaPpsbA-R | GCGATCACCGCTTCCCCCATGGTAAAATCTTGGTTTATTTAATTATCAGG |
| pBaTpsbA-F | CCAAGGTAGTCGGCAAATAAGACTTTGGTCTGATTGTATTGTATAGGA |
| pBaTpsbA-R | GAGCCCGCGGTGGCGGCCGCTAGTATAGTATTACTATATAATTTTTTTCCATTAAC |
| pBaPrrn-F | TCGACGGTATCGATAAGCTTTGCTACCCCGCCGTGAT |
| pBaPrrn-R | GCGATCACCGCTTCCCCCATAAATCCCTCCCTACAACGTATCCATGCGCTTCATATTCG |
| pBaTrbcL-F | CCAAGGTAGTCGGCAAATAACGTTCGTTCTCTTAATTGAATTTCA |
| pBaTrbcL-R | GAGCCCGCGGTGGCGGCCGCACAATATACAGGATGGGTAGAAAAAGTT |
| pBaTrps16-F | CCAAGGTAGTCGGCAAATAATCAAACAAAATTAAATTAAGGAAATAAAAAAAG |
| pBaTrps16-R | GAGCCCGCGGTGGCGGCCGCATGGAATCCATATAGAATAAAGAACTTCTATTC |

**Supplementary Table 3.** Comparison of *E. breviscapus* chloroplast genomes.

| **Region** | **Features** | **This article** | **Meng et al.** | **Li et al** |
| --- | --- | --- | --- | --- |
| LSC | Length (bp) | 84,657 | 84,705 | 84,881 |
|  | Length Percentage (%) | 55.6 | 55.7 | 55.7 |
| SSC | Length (bp) | 18,109 | 18,112 | 18,102 |
|  | Length Percentage (%) | 11.9 | 11.9 | 11.9 |
| IR | Length (bp) | 24,699 | 24,683 | 24,692 |
|  | Length Percentage (%) | 32.5 | 32.4 | 32.4 |
| Total | Length (bp) | 152,164 | 152,183 | 152,367 |
|  | GC Content (%) | 37.2 | 37.2 | 37.1 |

**Supplementary Table 4.** Genes with intron and their length of exons and introns in cp genome of *E. breviscapus*.

| **Gene name** | **Location** | **Exon I (bp)** | **Exon II (bp)** | **Exon III (bp)** | **Intron I (bp)** | **Intron II (bp)** |
| --- | --- | --- | --- | --- | --- | --- |
| *trnA-UGC* | IR | 37 | 36 |  | 820 |  |
| *trnE-UUC* | IR | 32 | 40 |  | 780 |  |
| *trnL-UAA* | LSC | 35 | 50 |  | 444 |  |
| *trnK-UUU* | LSC | 36 | 38 |  | 2543 |  |
| *clpP* | LSC | 71 | 294 | 226 | 817 | 622 |
| *ycf3* | LSC | 124 | 230 | 153 | 706 | 748 |
| *rps12* | LSC | 26 | 232 | 114 | 535 |  |
| *rpl2* | IR | 391 | 434 |  | 671 |  |
| *ndhA* | SSC | 553 | 539 |  | 1069 |  |
| *ndhB* | IR | 777 | 756 |  | 675 |  |
| *petB* | LSC | 6 | 642 |  | 801 |  |
| *atpF* | LSC | 145 | 410 |  | 709 |  |
| *rpoC1* | LSC | 430 | 1640 |  | 754 |  |

**Supplementary Table 5.** Statistics of codon usage bias.

| **Condon** | **Amino acid (aa)** | **% of aa** | **Number** | **RSCU** |
| --- | --- | --- | --- | --- |
| UAC | Y | 17.83% | 146 | 0.36 |
| UAU | Y | 82.17% | 673 | 1.64 |
| UGG | W | 100.00% | 391 | 1 |
| GUA | V | 37.46% | 442 | 1.5 |
| GUC | V | 11.86% | 140 | 0.47 |
| GUG | V | 14.58% | 172 | 0.58 |
| GUU | V | 36.10% | 426 | 1.44 |
| ACA | T | 29.50% | 331 | 1.18 |
| ACC | T | 18.63% | 209 | 0.75 |
| ACG | T | 10.70% | 120 | 0.43 |
| ACU | T | 41.18% | 462 | 1.65 |
| AGC | S | 5.82% | 97 | 0.35 |
| AGU | S | 20.50% | 342 | 1.23 |
| UCA | S | 19.90% | 332 | 1.19 |
| UCC | S | 15.71% | 262 | 0.94 |
| UCG | S | 8.51% | 142 | 0.51 |
| UCU | S | 29.56% | 493 | 1.77 |
| AGA | R | 29.85% | 391 | 1.79 |
| AGG | R | 10.84% | 142 | 0.65 |
| CGA | R | 22.75% | 298 | 1.36 |
| CGC | R | 6.49% | 85 | 0.39 |
| CGG | R | 6.95% | 91 | 0.42 |
| CGU | R | 23.13% | 303 | 1.39 |
| CAA | Q | 75.56% | 603 | 1.51 |
| CAG | Q | 24.44% | 195 | 0.49 |
| CCA | P | 28.80% | 271 | 1.15 |
| CCC | P | 17.64% | 166 | 0.71 |
| CCG | P | 15.41% | 145 | 0.62 |
| CCU | P | 38.15% | 359 | 1.53 |
| AAC | N | 23.72% | 241 | 0.47 |
| AAU | N | 76.28% | 775 | 1.53 |
| AUG | M | 100.00% | 510 | 1 |
| CUA | L | 13.06% | 307 | 0.78 |
| CUC | L | 6.34% | 149 | 0.38 |
| CUG | L | 6.51% | 153 | 0.39 |
| CUU | L | 22.54% | 530 | 1.35 |
| UUA | L | 30.28% | 712 | 1.82 |
| UUG | L | 21.27% | 500 | 1.28 |
| AAA | K | 73.98% | 799 | 1.48 |
| AAG | K | 26.02% | 281 | 0.52 |
| AUA | I | 30.22% | 551 | 0.91 |
| AUC | I | 20.24% | 369 | 0.61 |
| AUU | I | 49.53% | 903 | 1.49 |
| CAC | H | 25.19% | 133 | 0.5 |
| CAU | H | 74.81% | 395 | 1.5 |
| GGA | G | 37.77% | 590 | 1.51 |
| GGC | G | 11.91% | 186 | 0.48 |
| GGG | G | 18.18% | 284 | 0.73 |
| GGU | G | 32.14% | 502 | 1.29 |
| UUC | F | 35.42% | 425 | 0.71 |
| UUU | F | 64.58% | 775 | 1.29 |
| GAA | E | 72.86% | 827 | 1.46 |
| GAG | E | 27.14% | 308 | 0.54 |
| GAC | D | 20.00% | 184 | 0.4 |
| GAU | D | 80.00% | 736 | 1.6 |
| UGC | C | 28.92% | 72 | 0.58 |
| UGU | C | 71.08% | 177 | 1.42 |
| GCA | A | 29.06% | 365 | 1.16 |
| GCC | A | 16.32% | 205 | 0.65 |
| GCG | A | 11.54% | 145 | 0.46 |
| GCU | A | 43.07% | 541 | 1.72 |
| UAG | * | 21.43% | 12 | 0.64 |
| UGA | * | 19.64% | 11 | 0.59 |
