# Supplementary material for: Complete Chloroplast Genome Sequence of Erigeron breviscapus and Characterization of Chloroplast Regulatory Elements
Source: Front Plant Sci. 2021 Nov 25;12:758290. doi: 10.3389/fpls.2021.758290 (PMC8657942; doi:10.3389/fpls.2021.758290)
Supplement: Supplementary file 4 [file Presentation_1.pptx]

## Slide 1
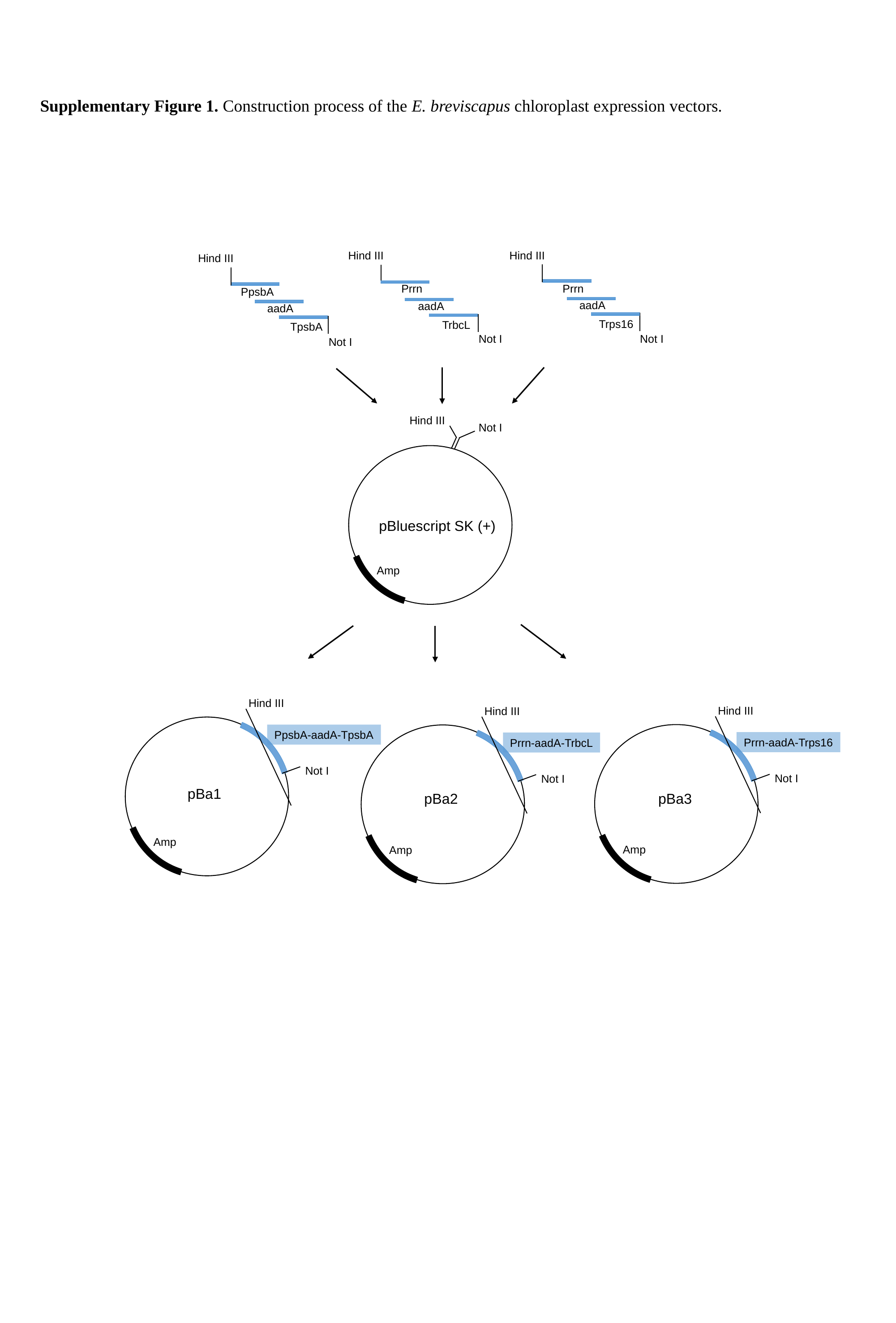

Supplementary Figure 1. Construction process of the E. breviscapus chloroplast expression vectors.
Hind III
Hind III
Hind III
Prrn
Prrn
PpsbA
aadA
aadA
aadA
Trps16
TrbcL
TpsbA
Not I
Not I
Not I
Hind III
Not I
pBluescript SK (+)
Amp
Hind III
Hind III
Hind III
PpsbA-aadA-TpsbA
Prrn-aadA-Trps16
Prrn-aadA-TrbcL
Not I
Not I
Not I
pBa1
pBa2
pBa3
Amp
Amp
Amp

## Slide 2
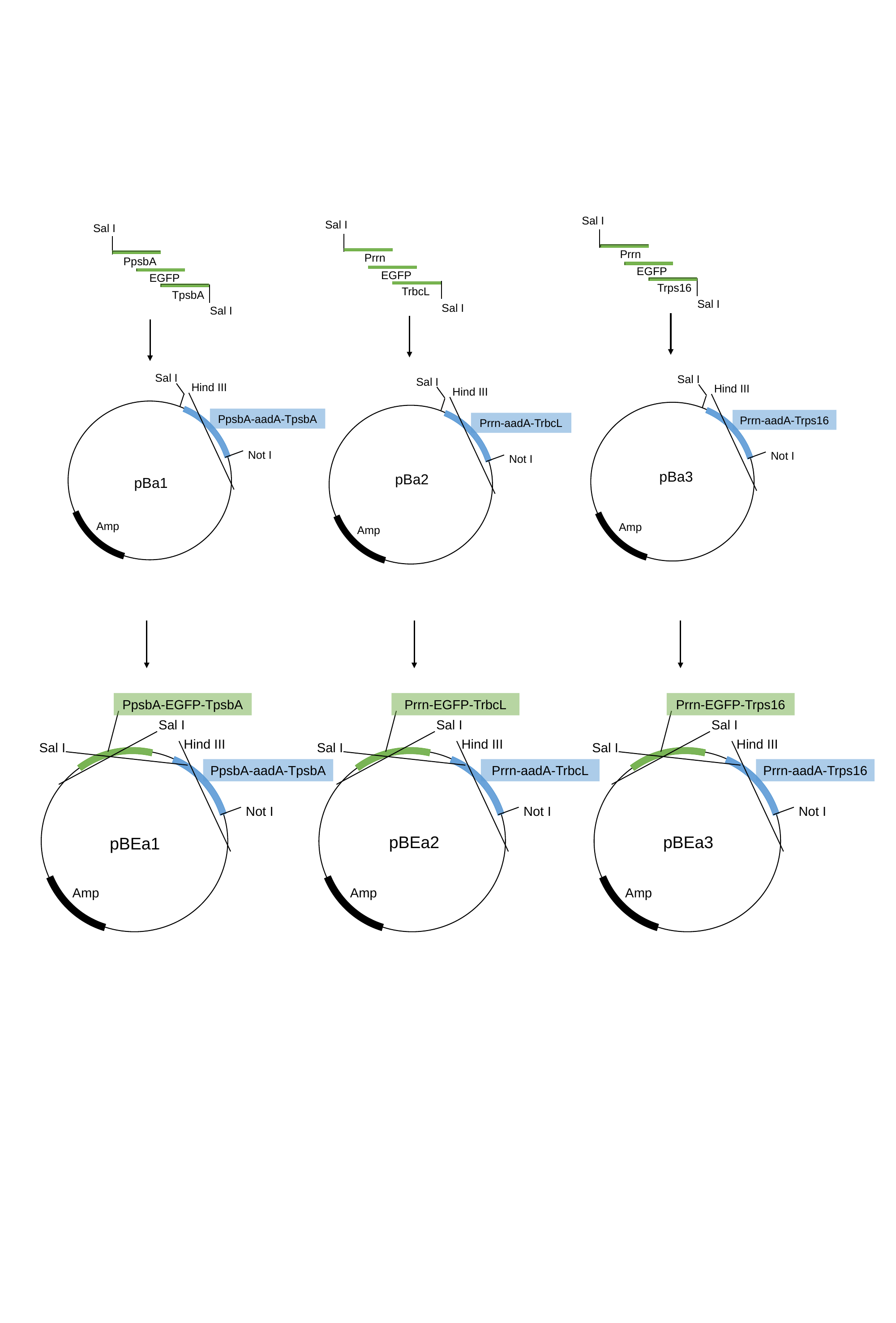

Sal I
Prrn
EGFP
Trps16
Sal I
Sal I
Hind III
Prrn-aadA-Trps16
Not I
pBa3
Amp
Sal I
Prrn
EGFP
TrbcL
Sal I
Sal I
Hind III
Prrn-aadA-TrbcL
Not I
pBa2
Amp
Sal I
PpsbA
EGFP
TpsbA
Sal I
Sal I
Hind III
PpsbA-aadA-TpsbA
Not I
pBa1
Amp
Prrn-EGFP-TrbcL
Prrn-EGFP-Trps16
PpsbA-EGFP-TpsbA
Sal I
Sal I
Sal I
Hind III
Hind III
Hind III
Sal I
Sal I
Sal I
Prrn-aadA-TrbcL
Prrn-aadA-Trps16
PpsbA-aadA-TpsbA
Not I
Not I
Not I
pBEa2
pBEa3
pBEa1
Amp
Amp
Amp

## Slide 3
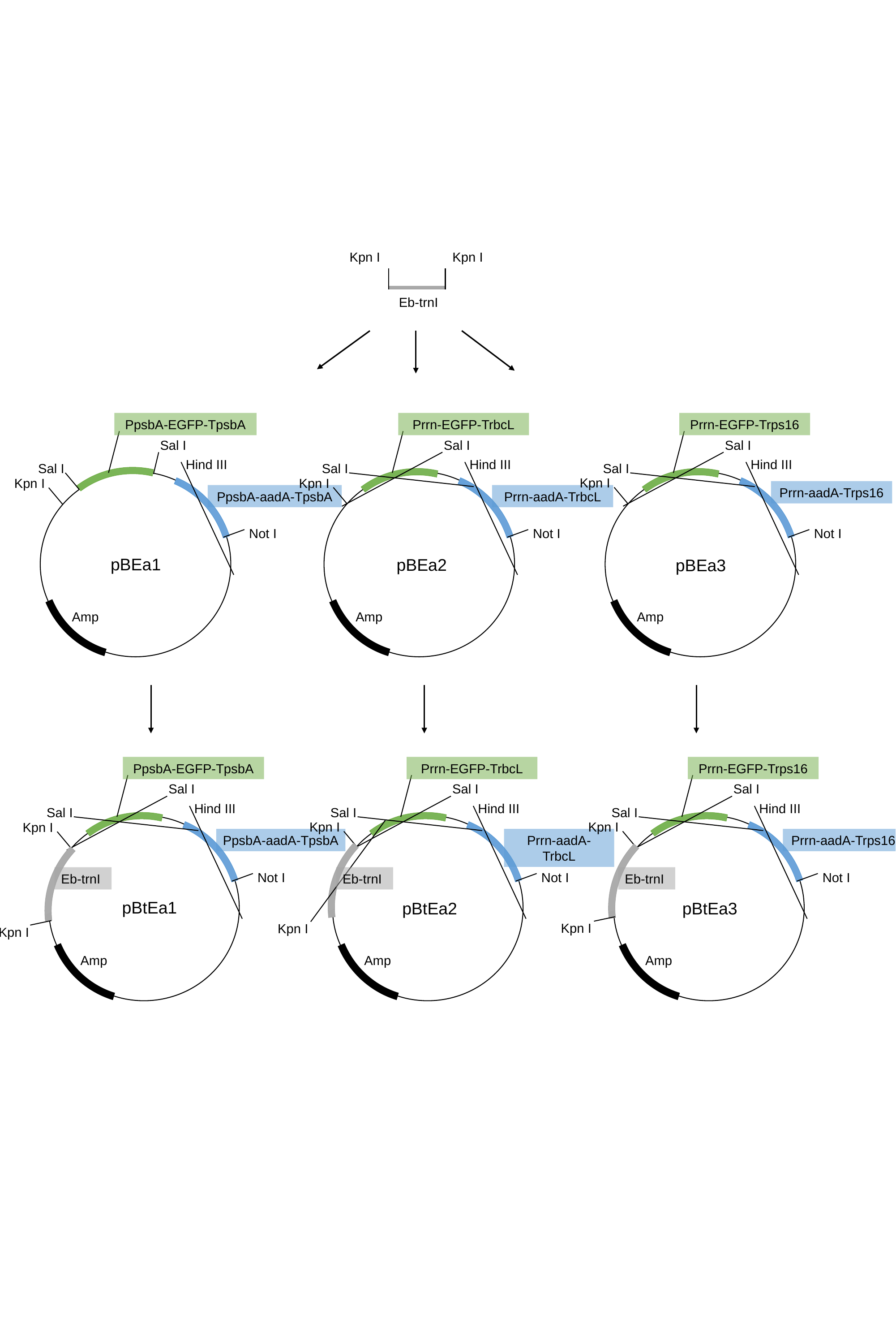

Kpn I
Kpn I
Eb-trnI
Prrn-EGFP-TrbcL
Prrn-EGFP-Trps16
PpsbA-EGFP-TpsbA
Sal I
Sal I
Sal I
Hind III
Hind III
Hind III
Sal I
Sal I
Sal I
Kpn I
Kpn I
Kpn I
Prrn-aadA-Trps16
Prrn-aadA-TrbcL
PpsbA-aadA-TpsbA
Not I
Not I
Not I
pBEa1
pBEa2
pBEa3
Amp
Amp
Amp
PpsbA-EGFP-TpsbA
Prrn-EGFP-TrbcL
Prrn-EGFP-Trps16
Sal I
Sal I
Sal I
Hind III
Hind III
Hind III
Sal I
Sal I
Sal I
Kpn I
Kpn I
Kpn I
PpsbA-aadA-TpsbA
Prrn-aadA-TrbcL
Prrn-aadA-Trps16
Not I
Not I
Not I
Eb-trnI
Eb-trnI
Eb-trnI
pBtEa1
pBtEa2
pBtEa3
Kpn I
Kpn I
Kpn I
Amp
Amp
Amp

## Slide 4
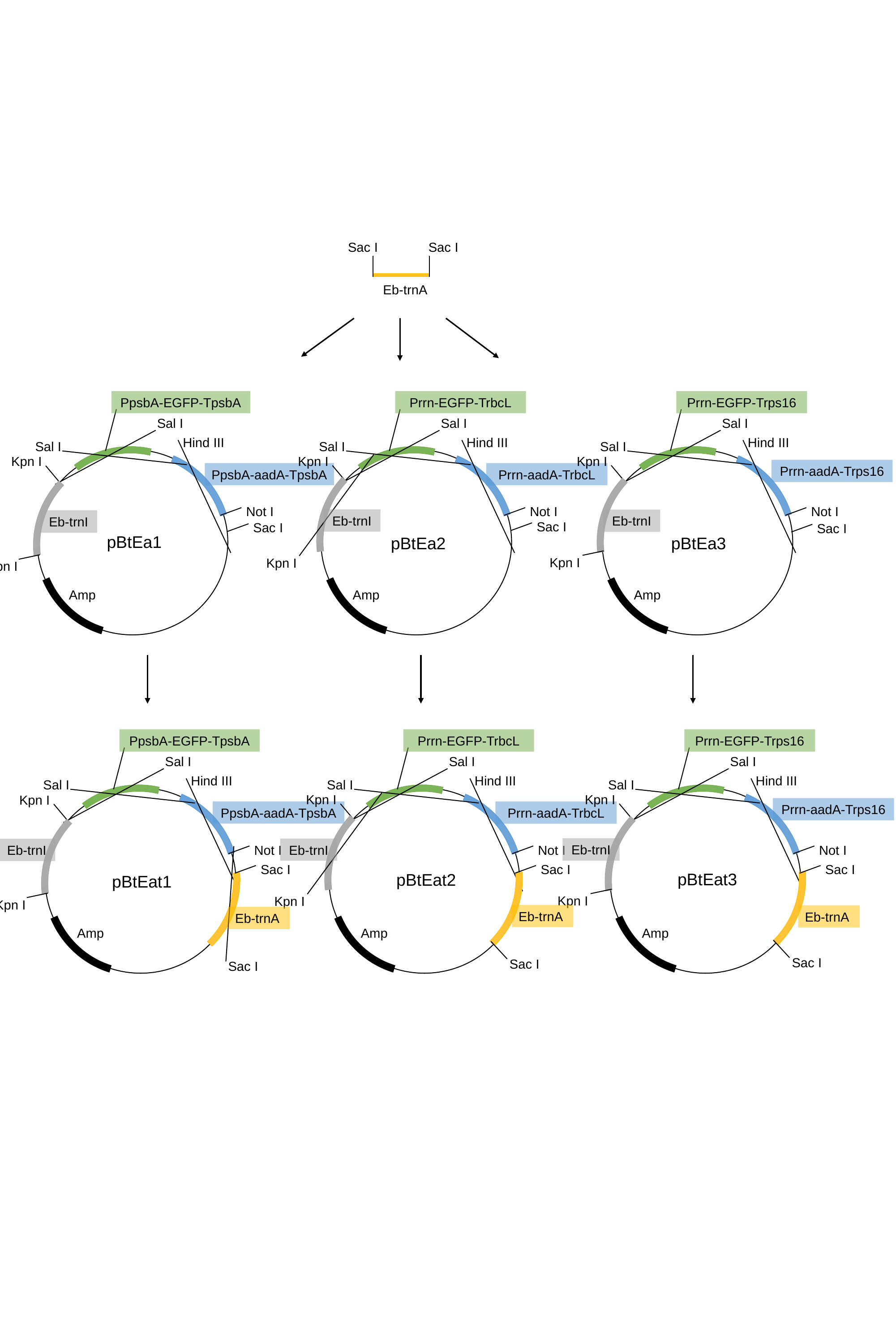

Sac I
Sac I
Eb-trnA
Prrn-EGFP-TrbcL
Prrn-EGFP-Trps16
PpsbA-EGFP-TpsbA
Sal I
Sal I
Sal I
Hind III
Hind III
Hind III
Sal I
Sal I
Sal I
Kpn I
Kpn I
Kpn I
Prrn-aadA-Trps16
Prrn-aadA-TrbcL
PpsbA-aadA-TpsbA
Not I
Not I
Not I
Eb-trnI
Eb-trnI
Eb-trnI
Sac I
Sac I
Sac I
pBtEa1
pBtEa2
pBtEa3
Kpn I
Kpn I
Kpn I
Amp
Amp
Amp
Prrn-EGFP-TrbcL
Prrn-EGFP-Trps16
PpsbA-EGFP-TpsbA
Sal I
Sal I
Sal I
Hind III
Hind III
Hind III
Sal I
Sal I
Sal I
Kpn I
Kpn I
Kpn I
Prrn-aadA-Trps16
PpsbA-aadA-TpsbA
Prrn-aadA-TrbcL
Eb-trnI
Eb-trnI
Not I
Not I
Not I
Eb-trnI
Sac I
Sac I
Sac I
pBtEat3
pBtEat2
pBtEat1
Kpn I
Kpn I
Kpn I
Eb-trnA
Eb-trnA
Eb-trnA
Amp
Amp
Amp
Sac I
Sac I
Sac I

## Slide 5
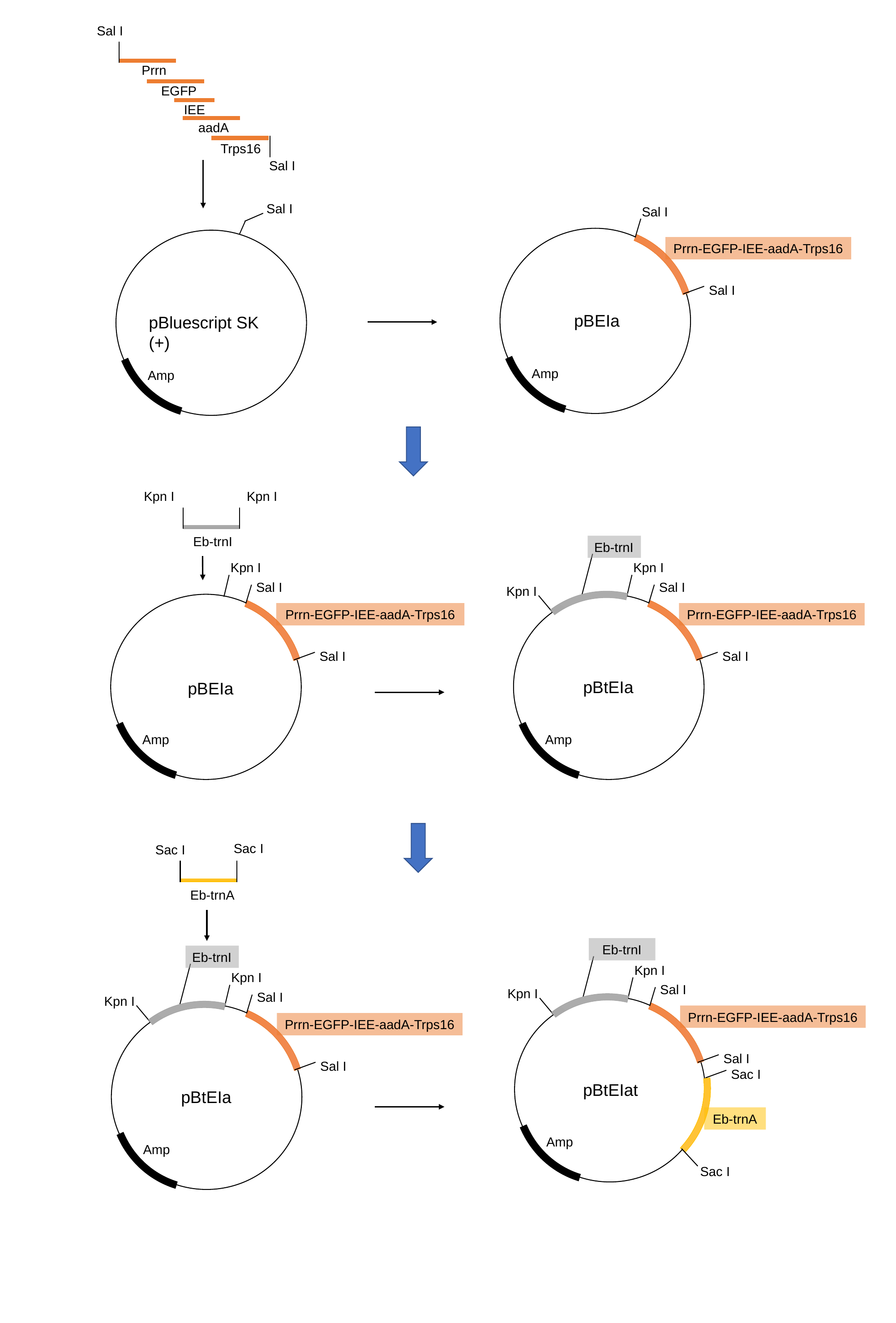

Sal I
Prrn
EGFP
IEE
aadA
Trps16
Sal I
Sal I
pBluescript SK (+)
Amp
Sal I
Prrn-EGFP-IEE-aadA-Trps16
Sal I
pBEIa
Amp
Kpn I
Kpn I
Eb-trnI
Kpn I
Sal I
Prrn-EGFP-IEE-aadA-Trps16
Sal I
pBEIa
Amp
Eb-trnI
Kpn I
Sal I
Kpn I
Prrn-EGFP-IEE-aadA-Trps16
Sal I
pBtEIa
Amp
Sac I
Sac I
Eb-trnA
Eb-trnI
Kpn I
Sal I
Kpn I
Prrn-EGFP-IEE-aadA-Trps16
Sal I
Sac I
pBtEIat
Eb-trnA
Amp
Sac I
Eb-trnI
Kpn I
Sal I
Kpn I
Prrn-EGFP-IEE-aadA-Trps16
Sal I
pBtEIa
Amp
